# New and Non-Official Remedies

**Published:** 1911-11

**Authors:** 


### New and Non-Official Remedies

Note.—This is a classified epitome of the report of the Council on Pharmacy and Chemistry of the A.M.A., descriptive of the articles accepted up to January 1, 1911. The first article in this series was published in our issue of October, 1911, and comprised new metallic compounds in which the metallic base itself is of therapeutic value.

SALICYLIC ACID COMPOUNDS.

Acid acetyl-salicylic (Aspirin). Claimed to pass stomach unchanged, to liberate salicylic acid gradually in intestine, hence to be relatively free from local and systemic by-effects. Dose 1-3—1 gram (5—15 grains).

Acid methylene citryl-salicylic. (Novaspirin) same claims.

Methyl benzoyl-salicylate (Benzosalin) same claims.

Succinyl disalicylic acid (Diaspirin) same claims.

Ethyl salicylate (Sal ethyl) analogous to methyl salicylate (oil of wintergreen) but less pungent.

Monoglycol salicylate (Spirosal) especially adapted to inunction, easily absorbed and not irritating. Full strength or diluted down to 1/8 with alcohol, oil, ointment base, etc.

Naphthol salicylate (Betol). Unchanged in stomach, split up into components in intestine. Intestinal and urinary antiseptic. Dose 30—50 centigrams (5—7½ grains).

Phenocoll salicylate. Antipyretic and analgesic action of phenocoll combined with that of salicylic acid. 1—2 grams (15—30 grs.)

Pyramidon salicylate, similar. 50—75 centigrams (7½—12 grains).

Hexamethylamine salicylate (Saliformin) urinary antiseptic and uric acid solvent. 1-3—2 grams (5—30 grains).

Acetyl paramidophenol salicylate. (Salophen) similar to salol but not toxic. 1-3—1 grams (5—15 grains).

Antipyrine salicylate. (Salipyrin) 1-3—2 grams (5—30 grains).

Methyl-oxymethyl salicylate (Mesotan, Ericin) analogous to oil of wintergreen but more irritating. Used mainly locally, 50 per cent. in olive oil.

ANTIPYRINE COMPOUNDS AND ANALOGUES.

Antipyrine salicylate (see above).

Antipyrine mandelate (Tussol) combines sedative and anti-pyretic action of antipyrine with glandular secretory stimulation. Especially recommended in pertussis. 30—50 centigrams (5—7½ grains) with regard to age.

Antipyrine and ferric chlorid (Ferri or Ferropyrine) 1-3—1 gram (5—15 grains).

Antipyrine Chloralhydrate. 1—2 grams (15—30 grains).

Dimethyl-amino-antipyrine. (Pyramidon). Claimed to be safer and more lasting but slower than antipyrine. 30—40 centigrams (5—6 grains).

Pyramidon acid camphorate.

Pyramidon neutral comphorate ½—1 gram (7½—15 grains).

Pyramidon salicylate (see under salicylic acid compounds).

Guaiacol salicylate (Guaiacol Salol) 1 gram (15 grains).

CHORAL COMPOUNDS.

Chloralformamid U.S.P. (Chloralamid).

Antipyrine chloralhydrate see under Antipyrine.

Chlorbutanol, tri-chlor-tertiary butyl alcohol. (Chloretone) (Methaform) antiseptic, local anaesthetic, especially in dentistry (saturated aqueous solution). Hypnotic and sedative in sea sickness, etc. 1-3—1½ grams (5—22 grains).

Butyl (or Croton) Chloralhydrate. Less depressing and more analgesic than chloral. 1-3—1 1-3 grams (5—20 grains).

BROMINE COMPOUNDS.

Acetone-bromoform. (Brometone) 30 centigrams (5 grains).

(Bromipin) Brominized sesame oil. 4—32 c.c. (1 drachm to 1 ounce) of 10 per cent. solution, other strengths proportionately.

Bromo-mangan about 33 parts of bromine in 10,000, combined with ferro-mangan q. v.

Bromural is 2-monobrom-isovaleryl-urea 1-3 gram (5 grains).

Bornyl-brom-valerate (Brovalol) 25—75 centigrams (4—12 grains).

Ethyl bromid (general anaesthetic by inhalation).

Calcium dibrom-behenate (Sabromin) dosage as for potassium bromid.

IODINE COMPOUNDS.

Calcium mono-iodo-behenate (Sajodin) 1—3 grams (15—45 grains).

Iodalbin, compound of iodine and blood albumin, containing 21.5 per cent. of iodine, decomposed mainly in intestine. 30—60 centigrams (5—10 grains).

Iodipin, iodized sesame oil 2—6 c.c. of 25 per cent. solution (30—90 minims) hypodermatically.

Iodoformogen, iodoform-albumin, nearly odorless, applied full strength.

Iodomangan. About 15 parts of iodine in 10,000 combined with ferromangan q. v.

Iodine, per-iodid of phthalic anhydride. Employed in oil, ointment, etc.

Iothion, di-iodo-hydroxy-propane. Employed externally in 25—50 per cent. lanolin ontment.

Iodo-chlor-hydroxy-quinolin (Vioform) (Nioform), antiseptic, hemostatic, cicatrizant. Employed as dusting powder, ointment, suppository, etc.

Iodothyrine. 1 gram equals 1 gram of fresh gland and contains 3—10 milligrams of iodine. Used mainly as thyroid extract. 1½—2 grams (7½—30 grains).

(Europhen) Di-iso-butyl-cresol-iodid. 20—30 centigrams (3—5 grains). Also externally as dusting powder, ointment, etc.

Thymol iodid U.S.P. (Aristol).

OXIDIZING COMPOUNDS ANALOGOUS TO HYDROGEN PEROXID.

Acetozone. Acetyl-benzoyl peroxid. 1:1000 of the powder (which is diluted 50 per cent. with an inert excipient). Drink ad lib. Also used in 1 per cent. mineral oil spray.

Succinic dioxid (Alphozone) used similarly.

(Perhydrol) 30 per cent. neutral solution of hydrogen peroxid.

Sodium perborate. Dusting powder or 2 per cent. solution. (Perogen, is sodium perborate mixed with a catalyzer, for preparation of oxygen baths).

Sodium peroxid. Not used internally. Mineral oil paste or soap used for acne, etc. (Oxone is sodium peroxid with a catalyzer).

Zinc peroxid used as dusting powder, ointment or soap.

CREOSOTE COMPOUNDS AND DERIVATIVES.

Anthrasol is a colorless coal tar, free from pyridin bases, etc., and mixed with juniper tar. Used externally, in ointment, etc.

Creosote carbonate (Creosotal) similar to creosote but claimed to be non-toxic and non-irritant.

Guaiacol coarbonate U.S.P. (Duotal).

Guaiacol benzoate (Benzosol) 20—60 centigrams (3—10 grains).

Guaiacol salicylate (Guaiacol-Salol) 1 gram (15 grains).

Glyceryl guaiacolate (Guaiamar) 1-3—1 1-3 grams (5—20 grains).

(Gujasanol) is diethyl-glycocoll-guaiacol hydrochlorid. 1—3 grams (15—45 grains). Subcutaneously 3—4 grams (45—60 grains) in 20 per cent. aqueous solution. Also in 1-10—2 per cent. ointment.

Guaiacol methyl-glycolate (Monotal) externally, analgesic and antipyretic, 2—4 c.c. by inunction.

Guaiacol cinnamate (Styracol) especially recommended for tuberculosis 1 gram (15 grains).

Potassium guaiacol sulphonate (Thiocol) 1-3—1 1-3 grams (5—20 grains).

Ethylene-diamine-trikresol (Kresamine) externally, mainly as disinfectant 2 per cent.

